# Repetitive Transcranial Magnetic Stimulation in Post-Herpetic Trigeminal Neuralgia: A Case Report in a 71-Year-Old Female Patient

**DOI:** 10.1192/j.eurpsy.2026.11754

**Published:** 2026-07-31

**Authors:** E. D. Cindik-Herbrueggen, A. S. Kucukali

**Affiliations:** 1 Neuro-Psychiatrisches Zentrum Riem, Munich, Germany; 2 Psychology, Istanbul Gelisim University, Istanbul, Türkiye

## Abstract

**Introduction:**

Trigeminal neuralgia (TN) is a debilitating neuropathic facial pain that profoundly impairs quality of life (Khawaja & Scrivani, *Dent Clin North Am* 2023; 67 99–115; Sveinsson et al., *Laeknabladid* 2025; 111 62–67). Post-herpetic TN, frequently observed in older adults after herpes zoster, often shows insufficient response to pharmacotherapy (Niemeyer et al., *Curr Pain Headache Rep* 2024; 28 295–306; Rohmayanti & Kurniawan, *J Pain Headache Vertigo* 2023; 4 1–6). rTMS over M1 has shown analgesic efficacy in chronic neuropathic pain (Wang et al., *Front Neurosci* 2023; 17 1158737; Dai et al., *Front Neurol* 2024; 15 1365445).

**Objectives:**

To report clinical application, tolerability, and preliminary outcomes of rTMS in an elderly female with chronic PHN-TN refractory to multiple treatments.

**Methods:**

A 71-year-old woman with >30-year left-sided TN after herpes zoster had daily pain. MRI excluded compressive lesion; EEG showed no epileptiform discharges. High-frequency rTMS over M1 (facial area): 10 Hz, 25–30 trains/session (100 pulses/train), 30-s inter-train interval, total 2,500–3,000 pulses, at 90–110% resting motor threshold; course 30–35 sessions with maintenance; 15 sessions completed at reporting. All identifiers removed and written informed consent was obtained.

**Results:**

Well tolerated; transient photophobia, mild balance disturbance; no serious adverse events. VAS pre/post each session; Sunnybrook for facial function; SF-36 for quality of life. Comparative analyses ongoing as treatment continues.

**Conclusions:**

rTMS appears feasible and safe for PHN-TN resistant to medication in an elderly patient, with preliminary signs of pain reduction and functional/quality-of-life improvement. Long-term follow-up and multidimensional outcomes (VAS, Sunnybrook, SF-36) are warranted to evaluate durability and effect size.

**Disclosure of Interest:**

None Declared

